# Supplementary material for: Peptide Engraftment on PEGylated Nanoliposomes for Bone Specific Delivery of PTH (1-34) in Osteoporosis
Source: Pharmaceutics. 2023 Feb 11;15(2):608. doi: 10.3390/pharmaceutics15020608 (PMC9965365; doi:10.3390/pharmaceutics15020608)
Supplement: Supplementary file 1 [file pharmaceutics-15-00608-s001.zip › pharmaceutics-2168308-SI.pdf]

Supplementary Material

Peptide Engraftment on PEGylated Nanoliposomes for Bone Specific Delivery of PTH (1-34) in Osteoporosis

Sagar Salave [1], Suchita Dattatray Shinde [1], Dhwani Rana [1], Bichismita Sahu [1], Hemant Kumar [1], Rikin Patel [2], Derajram Benival [1,*] and Nagavendra Kommineni [3,*]

1 National Institute of Pharmaceutical Education and Research (NIPER), Ahmedabad 382355, India
2 Intas Pharmaceuticals Ltd., Matoda Village, Ahmedabad 382210, India
3 Center for Biomedical Research, Population Council, New York, NY 10065, USA
* Correspondence: derajram@niperahm.res.in (D.B.); nagavendra.kommineni@gmail.com (N.K.)

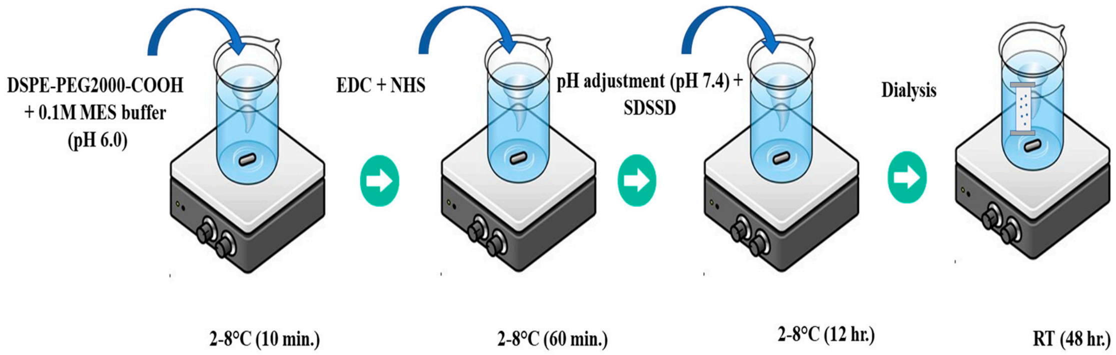

Figure S1. Diagrammatic representation for the synthesis of SDSSD-DSPE.

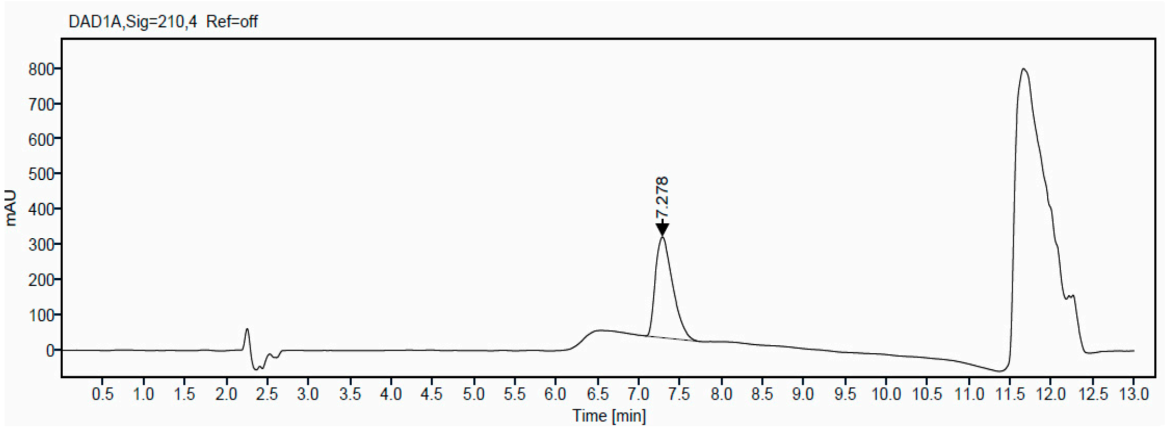

Figure S2. Standard chromatogram of SDSSD.

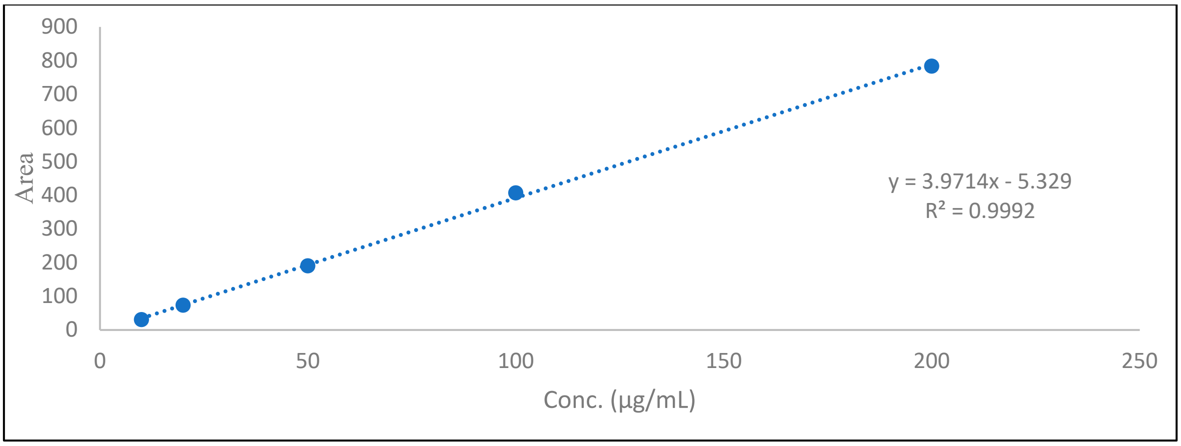

Figure S3. Linearity of SDSSD.

Table S1. The layout of the experimental design

Run no	Drug conc. (µM)	Lipid conc. (mM)	Cholesterol conc. (mM)	particle size (nm)	EE (%)
1	50.00	40.00	20.00	225.97	65.20
2	25.00	40.00	20.00	187.63	37.95
3	37.50	30.00	15.00	218.43	42.07
4	37.50	30.00	15.00	226.77	30.18
5	37.50	13.18	15.00	149.37	38.11
6	37.50	30.00	15.00	123.17	42.64
7	37.50	30.00	23.41	158.20	49.44
8	58.52	30.00	15.00	118.10	57.19
9	25.00	20.00	20.00	141.60	55.44
10	50.00	20.00	10.00	194.43	49.15
11	25.00	40.00	10.00	176.20	80.28
12	50.00	40.00	10.00	134.80	56.77
13	50.00	20.00	20.00	90.01	35.58
14	37.50	46.82	15.00	280.20	61.63
15	16.48	30.00	15.00	52.55	37.10
16	25.00	20.00	10.00	120.10	40.13
17	37.50	30.00	6.59	108.63	53.19

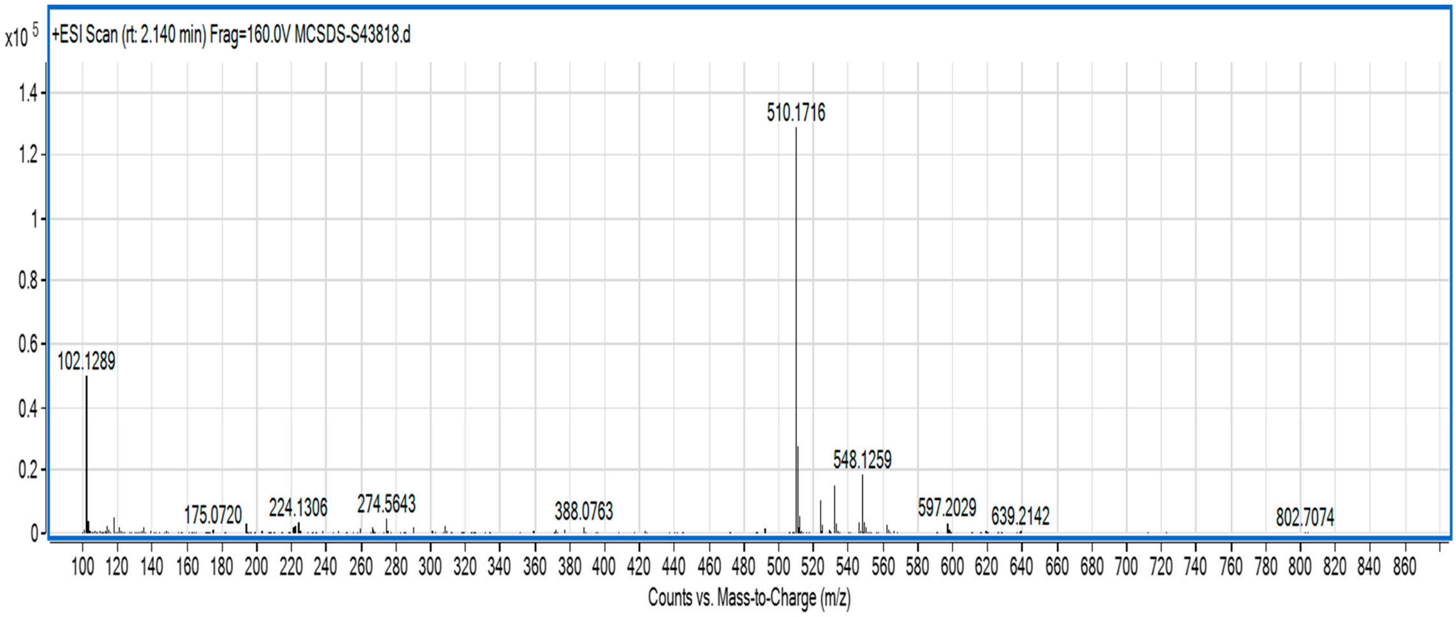

Figure S4. Mass spectra of SDSSD.

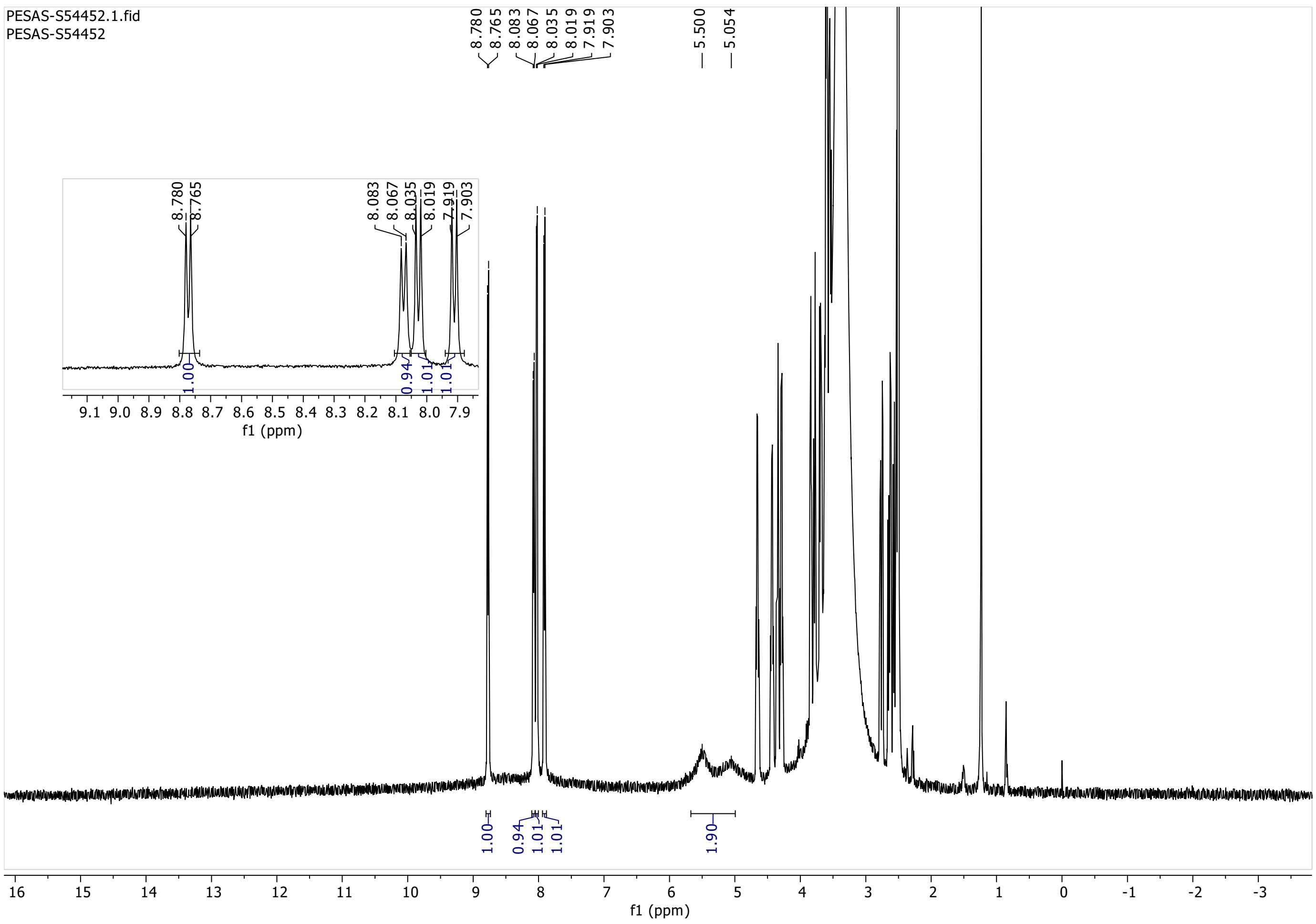

Figure S5. ^{1}H NMR of SDSSD in DMSO.

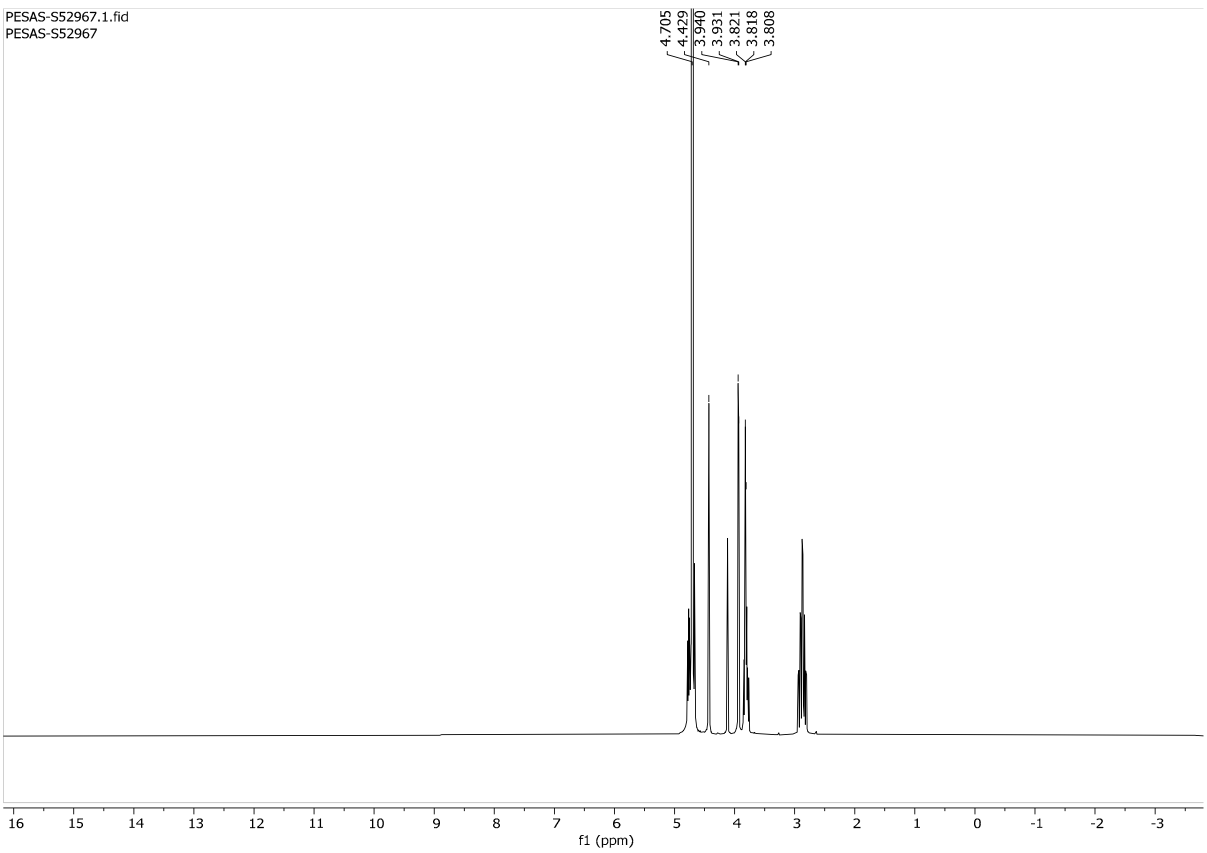

Figure S6. ^{1}H NMR of SDSSD in D_2O.

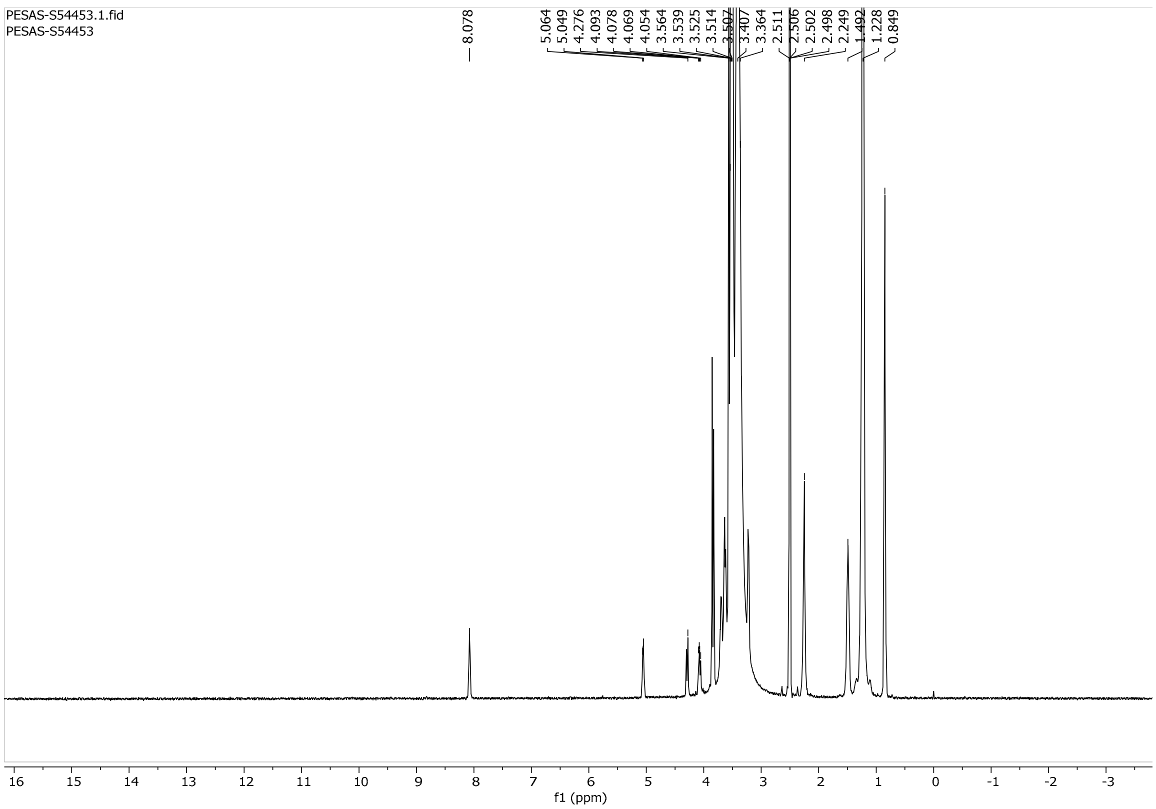

Figure S7. ^{1}H NMR of DSPE-PEG2000-COOH in DMSO

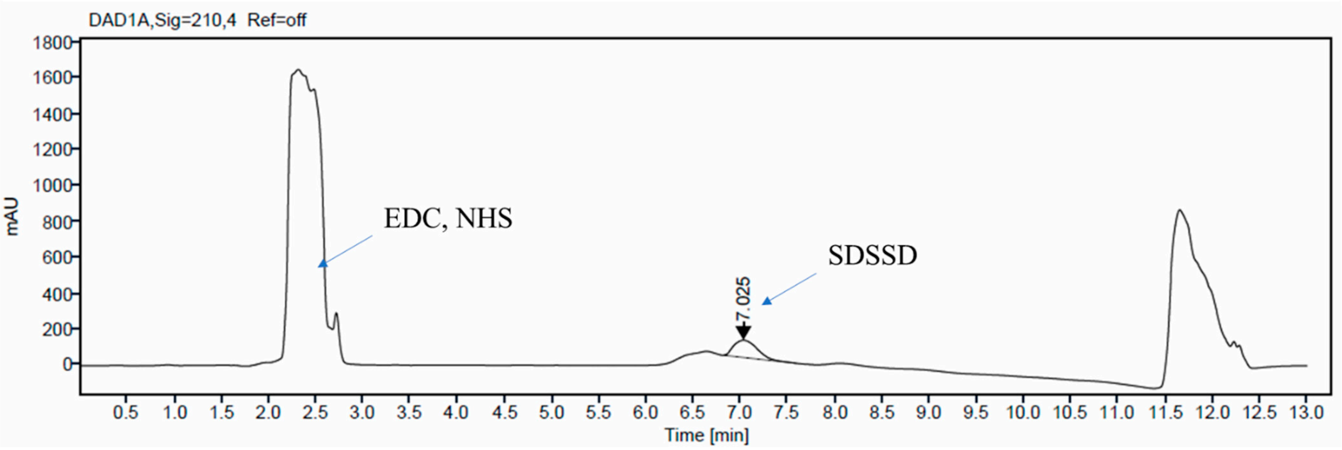

Figure S8. Determination of % conjugation efficiency of SDSSD-DSPE

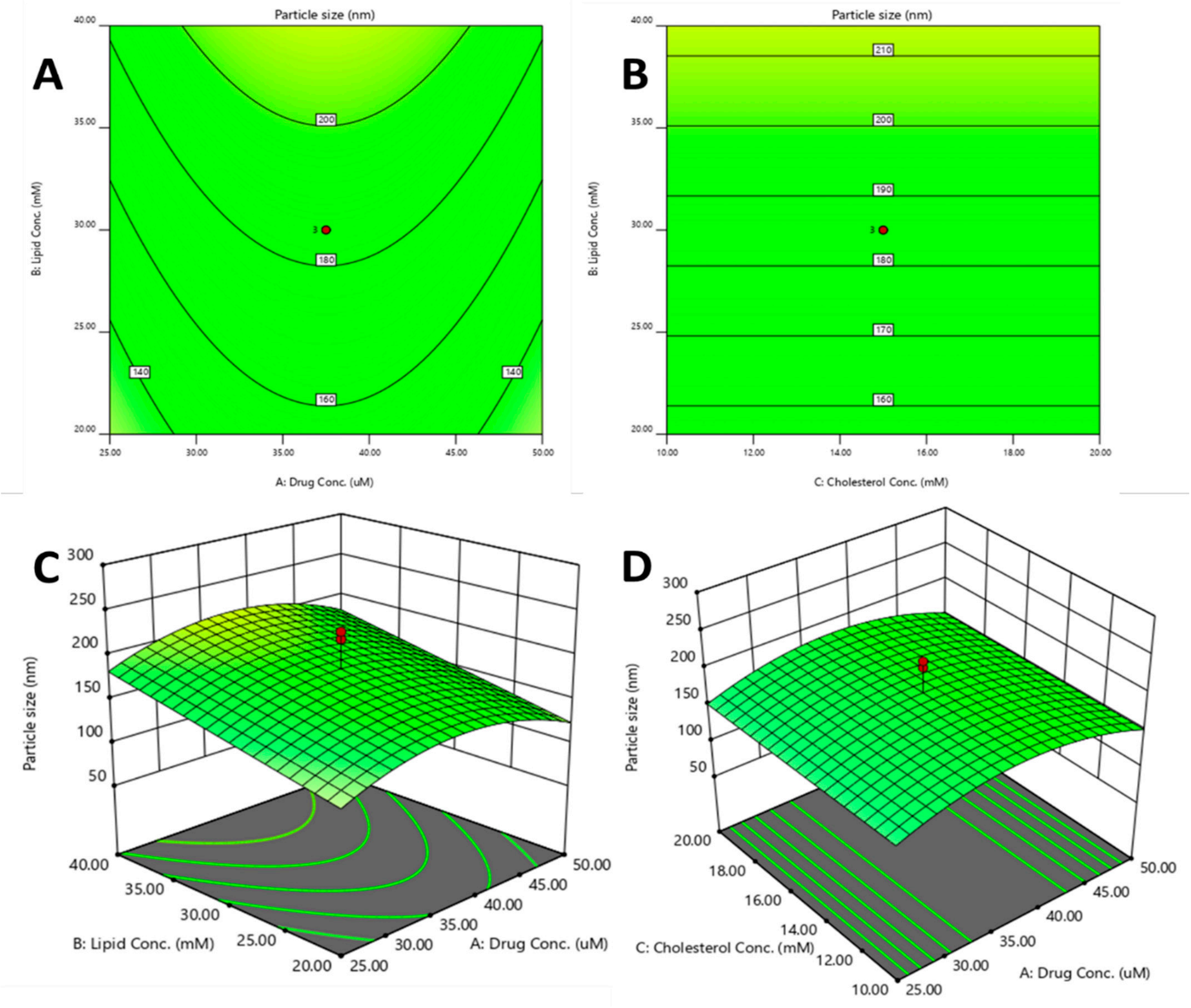

Figure S9. Graphical analysis for particle size. (A-B) Contour plot for particle size and (C-D) 3D surface plot for particle size.

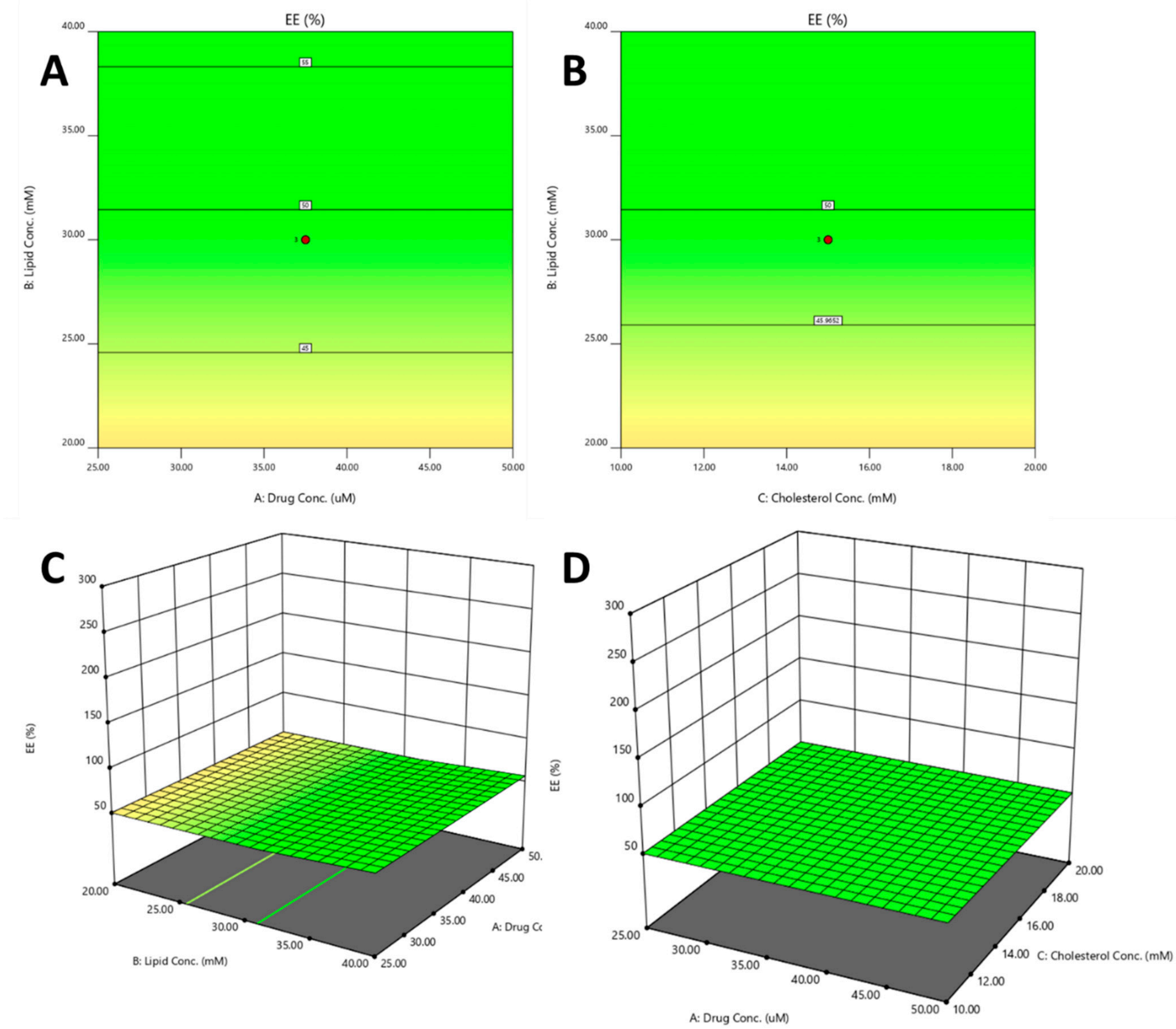

Figure S10. Graphical analysis for % EE. (A-B) Contour plot for %EE and (C-D) 3D surface plot for %EE.
